# Coarse-Grained Many-Body Potentials of Ligand-Stabilized Nanoparticles from Machine-Learned Mean Forces

**DOI:** 10.1021/acsnano.3c04162

**Published:** 2023-11-27

**Authors:** Giuliana Giunta, Gerardo Campos-Villalobos, Marjolein Dijkstra

**Affiliations:** Soft Condensed Matter, Debye Institute for Nanomaterials Science, Utrecht University, Princetonplein 5, 3584 CC Utrecht, The Netherlands

**Keywords:** Coarse-Graining, Computer Simulation, Machine Learning, Nanoparticles, Colloidal Systems, Self-Assembly

## Abstract

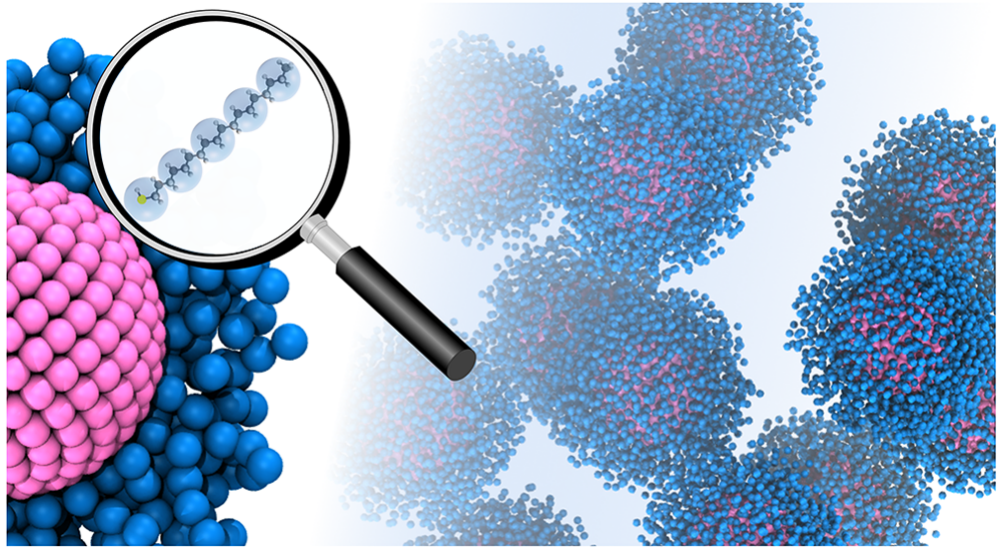

Colloidal nanoparticles self-assemble into a variety of superstructures with distinctive optical, structural, and electronic properties. These nanoparticles are usually stabilized by a capping layer of organic ligands to prevent aggregation in the solvent. When the ligands are sufficiently long compared to the dimensions of the nanocrystal cores, the effective coarse-grained forces between pairs of nanoparticles are largely affected by the presence of neighboring particles. In order to efficiently investigate the self-assembly behavior of these complex colloidal systems, we propose a machine-learning approach to construct effective coarse-grained many-body interaction potentials. The multiscale methodology presented in this work constitutes a general bottom-up coarse-graining strategy where the coarse-grained forces acting on coarse-grained sites are extracted from measuring the vectorial mean forces on these sites in reference fine-grained simulations. These effective coarse-grained forces, i.e., gradients of the potential of mean force or of the free-energy surface, are represented by a simple linear model in terms of gradients of structural descriptors, which are scalar functions that are rotationally invariant. In this way, we also directly obtain the free-energy surface of the coarse-grained model as a function of all coarse-grained coordinates. We expect that this simple yet accurate coarse-graining framework for the many-body potential of mean force will enable the characterization, understanding, and prediction of the structure and phase behavior of relevant soft-matter systems by direct simulations. The key advantage of this method is its generality, which allows it to be applicable to a broad range of systems. To demonstrate the generality of our method, we also apply it to a colloid–polymer model system, where coarse-grained many-body interactions are pronounced.

## Introduction

Metal and semiconductor nanocrystals (NCs) have attracted a lot of attention over the past few decades in many technological fields.^1^ NCs can self-assemble in a range of different two-dimensional and three-dimensional superstructures.^2−10^ Such nanostructured materials present an incredibly large surface-to-volume ratio, which makes them perfectly suited not only for optoelectronic, plasmonic, and photonic applications but also for catalysis, electrodes, and batteries. To prevent aggregation, NCs, such as PbSe, CdS, silver, and gold nanoparticles, are often protected with organic capping layers. For instance, in the case of gold nanoparticles, such molecules are often alkyl thiols.^7,11^ These ligand monolayers can undergo a temperature-dependent order–disorder transition in solvents like *n*-hexane or *n*-hexadecane, which switches the effective coarse-grained (CG) nanoparticle interactions from repulsive to attractive upon lowering the temperature.^11,12^ The self-assembly of nanoparticles is thus strongly dependent on the ligand type, the molecular solvent, and the temperature. In particular, the effect of the ligand shell on the mechanical and thermodynamic stability of such nanoparticles is not fully understood.^13^ A better theoretical understanding of the interactions between nanoparticles is thus of paramount importance for predicting the self-assembly process and the resulting structures. Atomistic simulations of ligand-coated nanocrystals may provide more insight but are severely limited by the length- and time-scales that can be achieved with present-day computers. Hence, computational studies of their phase and self-assembling behavior rely heavily on the use of simplified CG models based on effective CG interactions.

A common method employed to obtain information on the effective CG interactions of these complex nanoparticles is to perform potential mean force (PMF) calculations using computer simulations. In recent years, a substantial number of molecular simulation studies (including both Monte Carlo (MC) and molecular dynamics (MD) methods) on the effective CG pair interactions between alkanethiol capped nanocrystals in a vacuum and solvent have been reported.^3,7,14,15^ Nevertheless, while the effective CG pair interactions are the central focus and typical input of any many-body theory, much less is known about the effective CG triplet and higher-body interactions. Coarse-grained pair potentials are valid for large particle separations, but they break down for very high concentrations and short separation distances. The range of this effective CG pair potential involves a certain length scale, which may be, for instance, the decay length of the attractive van der Waals interactions, the Debye–Hückel screening length for charged colloids or twice the radius of gyration for star polymers.^16^

To have an estimate of the effect of many-body interactions, efforts have been paid to calculate effective CG three- and many-body interactions using nontrivial methods.^16−19^ Russ et al. have used nonlinear Poisson–Boltzmann theory to calculate the effective CG three-body interactions between charged colloids in a collinear, midplane, and equilateral triangle geometry.^20^ They found repulsive CG pair interactions and attractive CG triplet interactions. In the work of Von Ferber et al.,^16^ the authors have calculated by theory and computer simulations effective CG triplet interactions between star polymer centers in a good solvent positioned at the corners of an equilateral triangle. In both theory and simulations, they observed that the triplet contribution is weakly attractive even at short distances where the triplet overlap is substantial. However, while their theory can be extended for any triplet configuration, the investigation for arbitrary triplet configurations is computationally expensive. A similar approach has been employed in the work of Schapotschnikow and Vlugt^18^ where the authors performed MD simulations of interacting gold NCs in a vacuum for different sizes and varying ligand lengths (C_4_ to C_12_). By computing PMFs for pairs and triplets of NCs, they observed a strong attractive pair interaction due to the van der Waals contributions; the latter case resulted in a positive potential-energy correction of 20% and 40% in triplets consisting of short and long ligands, respectively. Interestingly, they gave estimates of the free energy for different configurations, triangles, and chains and found, in agreement with previous experimental studies, that for long capping molecules, the linear arrangement is energetically preferred over the triangle. The three-body contribution to the free energy has been estimated also in polymer-grafted nanoparticles (NPs) in a polymer matrix.^21^ To this end, Tang and Arya^21^ employed the so-called “blue moon ensemble” method. In such a method, constrained MD simulations are used for a test NP interacting with two NPs along a set of reaction coordinates differing in their orientation with respect to the NP-dimer axis. The three-body contribution was found to be repulsive and anisotropic, with the degree of repulsion increasing with the angular deviation from the NP-dimer axis. In a recent work, Liepold and coauthors^19^ proposed a method to study a pseudoatom model of dodecanethiol-ligated gold core nanoparticles in a vacuum, arranged on a square array with periodic boundary conditions to extract both the effective CG three- and four-body contributions. They found that the effective CG pair potential of the mean force in such a configuration is different from the isolated one. In particular, they observed that the combined three- and four-body contributions present an attractive well, implying that these many-body contributions are of comparable magnitude and opposite sign.

The computational works described above represent successful examples of approaches that have been used to obtain information on the effective CG many-body interactions between nanoparticles. However, accurately determining the scalar effective CG two- and three-body interactions of ligand-stabilized nanoparticles remains a formidable task as it requires the identification of suitable reaction coordinates for effectively integrating the gradients. In the case of a two-body potential of mean force, this can be accomplished by measuring the gradients of the potential of mean force (mean forces) on the nanoparticles at different distances *r* and integrating these forces along the reaction coordinate *r*. In the case of three-body potentials of mean force, one is often limited to specific configurations, such as an equilateral arrangement or a linear arrangement of the three particles. Furthermore, the functional dependence of the PMF on the internal coordinates can be quite complex to be represented by semiempirical functions, thus limiting their practical use in computer simulations.^22^ Despite extensive research in the field, full expressions of the effective CG three- or many-body interactions as a function of the coordinates of all nanoparticles have not yet been achieved.

In recent years, machine-learning (ML) approaches have been exploited for the construction of effective CG interaction potentials as a function of the local structure.^23,24^ The majority of these techniques have been developed to speed up *ab initio*-based MD simulations, where the energy and forces are not anymore directly evaluated every step via costly electronic structure calculations but instead represented by (generally nonlinear) functions of descriptors of local atomic environments.

More recently such methods have been successfully employed to represent effective CG many-body interactions in a variety of soft-matter systems such as spherical microgel particles in two dimensions,^25^ mixtures of colloidal hard spheres and rods with a nonadsorbing polymer,^26,27^ as well as two-body PMFs of ligand-coated rod-like particles and rod-like microgel particles.^27^ As demonstrated in these works, ML techniques are a powerful tool for speeding up simulations that consider CG many-body effects. It is important to stress that in the aforementioned systems, the local particle environments have been correlated to the CG interaction potential, which was in those cases a well-defined and accessible scalar function.

In 2017, Botu and co-workers^28^ introduced a ML approach based on a nonlinear association between atomic configurations and quantum-mechanical forces to construct ML force fields for elemental bulk solids with high chemical accuracy. CG many-body interactions of molecules, based on the relationship between atomic positions and mean forces, have also been developed by John and Csányi using Gaussian process regression.^29^ More recently, Gautham and Patra proposed a deep learning framework to learn the interactions between a pair of single-chain grafted spherical nanoparticles from their molecular dynamics trajectory,^30^ and Köller et al. have proposed a bottom-up coarse-graining method that combines classical force-matching with deep generative modeling, which has been shown to produce computationally efficient CG models that can capture the folding and unfolding transitions of small proteins.^31^

Here, we introduce a thermodynamic consistent coarse-graining approach, where we match the effective CG forces acting on CG sites with the vectorial mean forces as extracted from high-resolution simulations. In the case of typical truly force-based force fields, computing the (potential/free) energy, required for Monte Carlo simulations, necessarily relies on the definition of a pathway connecting the different configurations in phase space (either in time or along a reaction coordinate) in order to accurately carry out a force integration. This is a limitation of using a truly force-based force field, wherein, one cannot predict (potential/free) energies by simply choosing two arbitrary points in phase space.^28^ Our proposed ML strategy overcomes such a limitation as one can directly access the scalar effective CG many-body PMF without resorting to force integration or thermodynamic integration. To this end, we represent the effective CG forces, i.e., gradients of the potential of mean force or of the free-energy surface, by a simple linear model in terms of gradients of structural descriptors, which are scalar functions that are rotationally invariant. Due to the linearity of our model and the way we train it, we are able to directly access the analytical scalar CG many-body potential as a function of all nanoparticle coordinates without the need to introduce or identify suitable reaction coordinates to measure the gradients and to subsequently integrate them to obtain a scalar many-body potential of mean force. This enables us to construct effective CG many-body potentials for complex colloidal nanoparticles within a force-matching fashion. These effective CG many-body potentials can subsequently be employed in Monte Carlo simulations. We apply our approach to systems of ligand-stabilized nanoparticles in solvents of varying quality and demonstrate that the intricate effective CG potential of the mean force or free-energy landscape can be accurately represented by a simple linear ML model. Using the effective CG potential of mean force in Monte Carlo simulations, we demonstrate that the phase behavior and structure, as predicted by the ML model, are consistent with those exhibited by extensive molecular dynamics simulations of the fine-grained model.

Hence, the key result of our paper is that we obtain the effective CG many-body potential, which can directly be used in Monte Carlo simulations by machine learning the mean forces in fine-grained simulations without relying on any thermodynamic integration method. Our simple coarse-graining ML framework that we present is generic and extensible to other systems, and we expect that this approach will enable and accelerate simulation studies on the phase and self-assembling behavior of complex colloidal systems—by overcoming spatiotemporal limitations of fine-grained models.

## Results and Discussion

### Fine-Grained Model of Ligand-Stabilized Nanoparticles

**Figure 1:**
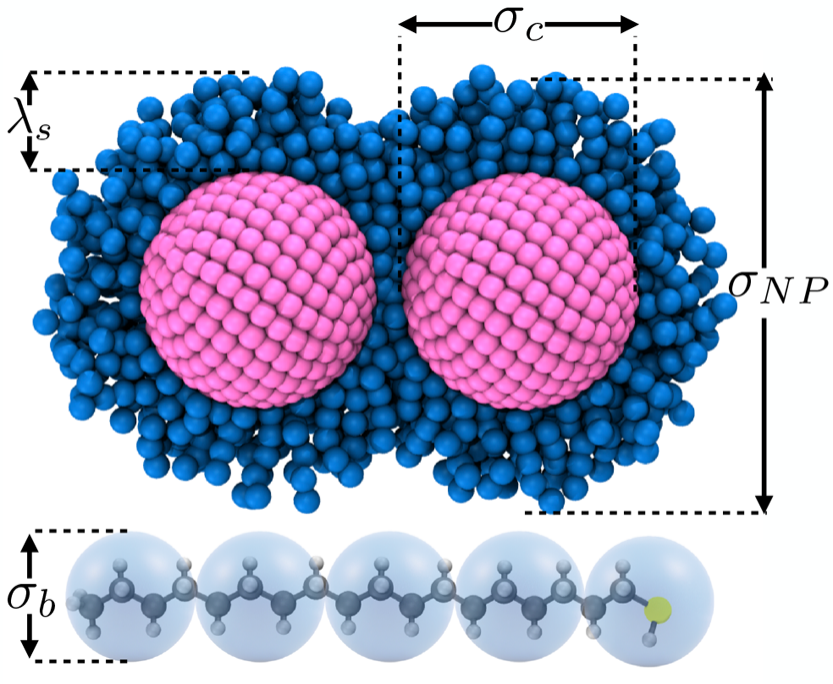
Schematic representation of two nanoparticles with a hard core of diameter σ_c_ and capped with a soft-deformable shell of covalently anchored ligands with a thickness of approximately λ_s_ such that the effective diameter of each nanoparticle is approximately σ_NP_ = σ_c_ + 2λ_s_. The blue spheres correspond to MARTINI ligand beads, and the pink beads represent the nanocrystal core. Note that in order to highlight the core–corona architecture of the particles, only ligand chains on a hemisphere of the NP cores are shown. In the fine-grained representation adopted here, a single surface-attached ligand (octadecanethiol) consists of 5 MARTINI beads of diameter σ_b_, which represent 18 carbon atoms.

We start by introducing the high-resolution model of ligand-stabilized nanoparticles (NPs), which we will refer to as the “fine-grained” (FG) model. Here, we adopt a detailed representation of the core–corona NPs based on the MARTINI force field, suited for molecular dynamics simulations of macromolecules, such as polymers, copolymers, surfactant molecules, sugars, and a variety of nanoparticles.^32,33^ In particular, the ligands, covalently bonded to the cores, are represented as chains of 5 “C1”-type MARTINI beads, approximately corresponding to alkyl ligands of 18 carbon atoms and a headgroup (e.g., thiol or amine). NP cores are modeled as rigid bodies with a spherical shape of diameter σ_c_ = 4.2 nm and consist of *n*_c_ = 275 core beads depicted in pink in Figure 1. For an isolated NP, the effective thickness of the floppy capping layer of ligand λ_s_ depends on the solvent quality and can be estimated from a radial density profile with its origin in the nanocrystal core. Thus, the fully capped NPs can be characterized with an incompressible hard core of diameter σ_c_ and an effective partially compressible soft diameter of σ_NP_ = σ_c_ + 2λ_s_ (see Figure 1). A similar representation has been recently employed to model ligand-stabilized nanorods.^27^ The surface coverage, calculated as the number of ligands per surface area, is 5 nm^–2^.^13^ Hence, each NP is covered by 275 ligands, corresponding to *n*_l_ = 1375 ligand beads per NP, and thus, each NP in the FG model consists of *n*_b_ = *n*_c_ + *n*_l_ = 1650 beads in total. To mimic static effects of a solvent in an implicit fashion, we follow the approach by Fan et al.,^34^ where pair interactions between nonbonded beads are modeled through a modified Lennard-Jones (LJ) potential by the introduction of a “weight” parameter *s* that controls the strength of the pair interactions relative to the original MARTINI value (see Methods). In particular, we use two values of *s* here, namely *s* = 0.1 and *s* = 0.3, in order to model “good” and “bad” solvent conditions, respectively. We note that the limiting case of *s* = 1 recovers the original LJ potential, which mimics an extremely bad solvent (or vacuum) for the NPs, while a value of *s* = 0 corresponds to a fully repulsive Weeks–Chandler–Andersen pair interaction, leading to purely steric interactions between NPs. The adopted implicit solvent representation may omit some features on the effective interactions between NPs. However, Fan et al.^34^ demonstrated in their study that the potentials of mean forces obtained from simulations using the implicit solvent representation compared well with those obtained from an explicit solvent model, thereby justifying the implicit solvent approach. In addition, we fixed the temperature *T* = 300 K of the nanocrystal suspension.

### Thermodynamic Consistent Coarse-Grained Model

Following the description of the FG model, a given configuration of a collection of *N* nanoparticles, each comprising of *n*_b_ beads, is represented by the positions of *n* = *n*_b_*N* beads ***r***^*n*^ = {***r***_1_, ..., ***r***_*n*_}, . The probability density of finding a certain configuration ***r***^*n*^ in the canonical ensemble reads *p*_FG_(***r***^*n*^) ∝ exp[−*βϕ*(***r***^*n*^)], where ϕ(***r***^*n*^) denotes the potential energy of configuration ***r***^*n*^ in the FG system, β = 1/*k*_B_*T* the inverse temperature, and *k*_B_ the Boltzmann constant. In contrast, the CG representation that we aim at obtaining here consists only of *N* sites with positions ***R***^*N*^ = {***R***_1_, ..., ***R***_*N*_}, . Hence we are dealing with the dimensionality-reduction problem: , which projects FG states ***r***^*n*^ onto a lower-dimensional representation ***R***^*N*^. This conversion is achieved by mapping ***R***^*N*^ = *M*(***r***^*n*^) that defines the CG coordinates in terms of the FG model configuration. The mapping function *M* can be an arbitrarily complex function of the coordinates of the *n* beads but is often defined as a simple linear transformation ***R***^*N*^ = ***Mr***^*n*^ with ***M*** the matrix of mapping coefficients. In the case that the mapping coefficients are only ones and zeroes, the CG coordinate corresponds to the centers of mass of the respective groups of beads. In this paper, we have taken the *N* CG sites to correspond to the centers of mass of the *N* nanocrystal cores by mapping the *n*_c_ core beads of a NP in the FG model onto the corresponding CG site in the CG representation.

Obtaining a thermodynamic consistent CG model defined through the mapping function above demands that the probability distribution of a CG configuration ***R***^*N*^ is the same for the CG model as for the high-resolution FG model.^35−37^ The probability distribution in the CG space depends on the effective CG interaction potential, Φ(***R***^*N*^) and reads *P*_CG_(***R***^*N*^) ∝ exp[−βΦ(***R***^*N*^)]. Using the probability density of the FG system and the mapping as described above yields the following probability density for the CG variables in the FG system:
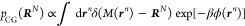
1Equating *P*_CG_(***R***^N^) and *p*_CG_(***R***^N^), we find that the thermodynamic consistent coarse-grained potential reads
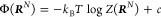
2where *Z*(***R***^*N*^) = *∫* d***r***^*n*^ δ(*M*(***r***^*n*^) – ***R***^*N*^) exp[−*βϕ*(***r***^*n*^)] and *c* is an arbitrary constant.

By definition, the gradient of Φ(***R***^*N*^) determines the effective CG force on CG site *I*
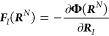
3Using eq 2 and the center-of-mass linear mapping from the *n* FG beads to the *N* CG sites as described above, the effective CG force on CG site *I* can be related to the mean force on CG site *I* in the FG representation
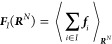
4where the angular brackets denote an ensemble average where the centers of mass of the *N* CG sites are kept fixed
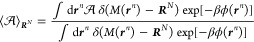
5and where ***f***_*i*_ denotes the instantaneous force on the *i*th FG bead belonging to CG site *I*. In addition, the summation denotes a sum over all of the forces on the FG beads composing the CG site *I*.

These mean forces can be efficiently sampled from constrained or restrained simulations of the FG model,^38^ allowing thus for a direct determination of the effective CG interaction potential Φ(***R***^*N*^) by integration of the gradients of the PMF along a predefined reaction coordinate. This is a typical approach that has been widely followed to compute effective CG pair interactions of nanoparticles,^7,18,39^ where mean forces are collected for a series of pair distances and subsequently integrated over that internal variable to obtain a scalar function approximating the underlying effective CG two-body PMF.

Equations 3 and 4 formally define the effective CG interaction potential Φ(***R***^*N*^) as a function of a constrained average over the FG model. However, it is clear that they simply set a direct relationship between both representation levels but do not provide a closed mathematical form for the CG potential Φ(***R***^*N*^) in terms of the CG coordinates that can be used without the need to resort to simulations of the FG model in each step. Thus, the practical challenge is to determine an explicit function Φ(***R***^*N*^) of the CG coordinates ***R***^*N*^ that represents the true CG many-body PMF. In this context, two practical methods have been developed to approach thermodynamic consistency while retaining tractable functional forms to serve as the CG potential: variational force-matching^40^ and relative entropy minimization.^41^ The latter approach and the closely related method known as iterative Boltzmann inversion (IBI)^42^ are data-efficient as they simply require structural sampling (for example, IBI sets a model where parameters are optimized so as to match distributions of the FG model); however, they require the CG model to be resimulated during the iterative training procedure, which can be extremely costly and even lead to failure in convergence.^31^ In contrast, force-matching is straightforward to implement but slightly more data-inefficient, as it requires the vectorial forces on the CG particles mapped from FG sampling as in eq 4. Here, we follow such an approach and focus on learning the mean forces sampled from constrained simulations of the FG model using linear models that employ vectorial basis functions describing the local structure in the mapped CG representation. We decide to follow this “multiscale force-matching” route as it has already been shown in recent developments on atomic ML potentials that the quantum-mechanical vectorial force acting on a particular atom, can be accurately learned and predicted directly from a configuration of atoms.^28,29^

Among the different ML potential variants, those based on Behler and Parrinello symmetry functions (SFs) have been widely used for constructing ML potentials for atomistic systems and more recently, also for colloidal and nanoparticle systems^26,27,43^ (see Descriptors: Symmetry Functions and their Gradients). The main idea in such an approach is to represent the total CG (potential or free) energy Φ(***R***^*N*^) of a system as a sum of per-atom (bead, particle, etc.) contributions Φ_*K*_, Φ = ∑_*K* = 1_^*N*^Φ_*K*_, where each individual contribution to the potential is in turn a function of a set of *N*_s_ SFs; Φ_*K*_ = Φ_*K*_({*G*_1_(*K*), ..., *G*_*N*_s__(*K*)}), which describe the local atomic environments. Hence, under such a construction, the αth component of the mean force *F*_*I*,α_ acting on CG site *I* with respect to coordinate *R*_*I*,α_ with α = (*x*, *y*, *z*) can be represented (by applying the chain rule) as
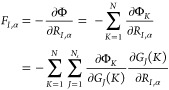
6where *G*_*J*_(*K*) is the *J*th SF in the set of *N*_s_ symmetry functions describing the local environment of CG site *K*. Note that the term ∂Φ_*K*_/*∂G*_*J*_(*K*) is fully determined by the regression method employed to construct the relationship between the effective interaction potential and the structure of the system (Φ_*K*_ = Φ_*K*_({*G*_1_(*K*), ..., *G*_*N*_*s*__(*K*)})). Based on previous works,^25−27^ we assume a simple linear relationship  such that eq 6 gives
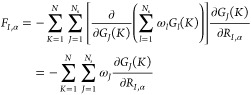
7

Strictly speaking, the many-body PMF is defined as a difference with respect to a reference state, and a numerical constant *c* would typically appear (see eq 2). However, when the reference point is defined as the state ***R***^*N*^ at infinite dilution (essentially an ideal gas), such a constant is identical to zero. This means that the training set should contain enough low-density configurations where the mean forces vanish in the FG representation. Due to the construction of the PMF, this accounts to conceiving each per-particle potential contribution as a difference with respect to the case when they are isolated. By simply using the weights ω_*J*_ and the analytical derivatives of SFs *∂G*_*J*_(*K*)/*∂R*_*I*,α_, the mean forces on the CG particles can be predicted and employed straightforwardly in MD simulations, where only forces are needed to propagate the system. An additional advantage of our approach is evident from eq 7, which establishes a simple and direct way to obtain an effective many-body CG interaction potential Φ(***R***^*N*^) for a system of *N* nanoparticles by simply learning the mean forces (extracted, for example, from constrained simulations of the FG model). More specifically, given the linearity of the model, the effective many-body CG potential is readily obtained:
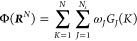
8

We mention here that the chosen fingerprint representation for describing the components of the particle forces in terms of derivatives of the SFs conforms with the required invariance operations, such as permutation, translation, and rotation of particles. For instance, consider a reference particle *I* and its neighboring atoms within a radial cutoff distance. Information pertaining to neighboring particles of particle *I* are passed into the summands of the individual SFs as scalar pairwise distances or angles; therefore, permutation or rigid translation of particles does not alter *G*_*K*_(*I*). In the case of rigid rotations, both the fingerprint ∇*G*_*K*_(*I*) and vectorial force components ***F***_*I*_ transform in an identical manner governed by the rotation matrix. Based on the premises above, the first step for a consistent coarse-graining of a system of *N* nanoparticles is the generation of a set of configurations in the FG representation using simulations of the FG model. Ideally, the configurations should be diverse enough that a *sufficient* number of different local particle environments are considered. Each of the FG configurations is then used as the initial state of subsequent simulations, where the centers of mass of the nanoparticle cores are frozen in order to efficiently measure the mean forces in eq 4. The vectorial mean forces are then fitted by simple linear regression using eq 7 with the feature selection method proposed in ref (25) (see Fitting Procedure).

### CG Two-Body Potential from ML Mean Forces

In order to test the proposed methodology, we first construct a simple linear model for the effective CG two-body forces, which are gradients of the true effective CG two-body PMF Φ^(2)^. For a direct test of the accuracy of the method, we first compute the effective CG two-body PMF Φ^(2)^ as a function of the separation distance *R*_*IJ*_ = |***R***_*I*_ – ***R***_*J*_| between two NPs using constraint MD simulations of the FG model.^38^ For each PMF calculation, we perform simulations with the NC nanoparticle cores frozen at 250 different distances *R*_*IJ*_. For each of these simulations, the two-body mean force *F*_m_(*R*_*IJ*_) is calculated as the average force between the two nanoparticle cores in the direction of their center-of-mass distance vector **R**_*IJ*_(18)
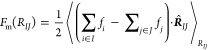
9where *∑*_*i*∈*I*_*f*_*i*_ and *∑*_*j*∈*J*_*f*_*j*_ denote a sum over all the instantaneous forces on the FG beads composing the nanoparticle core *I* and *J*, respectively, and  is the unit vector connecting the two nanoparticles along the reaction coordinate *R*_*IJ*_. Angular brackets denote ensemble averages in the canonical ensemble with constraint separation distance *R*_*IJ*_. The effective CG PMF Φ^(2)^(*R*_*IJ*_) is then directly computed by integration
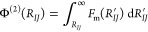
10

**Figure 2:**
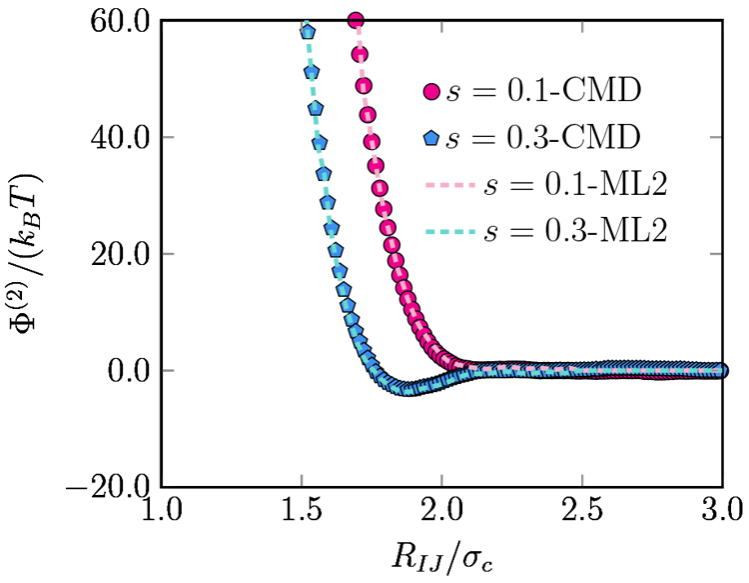
Coarse-grained two-body potential of mean force (PMF) Φ^(2)^ for a pair of nanoparticles with core diameter σ_c_ = 4.2 nm as a function of the center-of-mass distance of the two nanoparticle cores *R*_*IJ*_ for two different solvent qualities, *s* = 0.1 (good solvent) and 0.3 (bad solvent). Filled symbols correspond to the PMF values obtained from integrating the mean forces measured in constrained MD simulations of the FG model, whereas the dashed lines correspond to the values predicted by the potential constructed by directly learning the mean forces on the two particles (ML2).

We report the results of the effective CG two-body PMF Φ^(2)^(*R*_*ij*_) as obtained from the constrained MD simulations of the FG model for a solvent of *s* = 0.1 and *s* = 0.3 as filled symbols in Figure 2, which clearly show that the effective CG interactions between the nanoparticles are strongly influenced by the affinity of the ligands with the solvent. More specifically, when the solvent parameter is *s* = 0.1 (good solvent conditions), the interactions are purely repulsive, while when the solvent has a lower quality in the case of *s* = 0.3, the interactions become attractive at short distances. In particular, for *s* = 0.3 we find a minimum of −3.3 *k*_B_*T* at *R*_*IJ*_/σ_c_ = 1.88. At shorter distances, the interactions are repulsive in both cases, signaling the unfavorable compression of ligand chains. Interestingly, we notice that while the distribution of the ligand beads around the cores of isolated NPs in solvents with *s* = 0.1 and 0.3 is rather similar, the underling effective CG two-body interactions are significantly different.

Using the same setup of the constrained MD simulations, we prepare training data sets containing solely the vectorial components of the mean forces acting on the centers of mass of the two individual nanoparticle cores *I* and *J* for each separation distance (a total of 750 force components per particle). We proceeded to learn the mean forces using the scheme described above (see eq 7 and Fitting Procedure). The fitting is performed using only gradients of two-body SFs (see Descriptors: Symmetry Functions and their Gradients). The number of descriptors is *N*_SF_ = 46 and *N*_SF_ = 38 for *s* = 0.1 and *s* = 0.3, respectively. We use a single cutoff value of *R*_c_ = 2.5σ_c_. The ML models constructed with the gradients of the SFs present a correlation coefficient *R*^2^ ≈ 0.999 for the two solvents and root mean square error (RMSE) values of 14.78*k*_B_*T*/σ_c_ and 12.80*k*_B_*T*/σ_c_ for the solvents *s* = 0.1 and 0.3, respectively. Using the optimized weights (linear coefficients) of the gradients of the SFs and following eq 8, we obtain the effective CG two-body ML potentials Φ_ML2_^(2)^(*R*_*IJ*_) for 100 different configurations, where we place the CG nanoparticle cores at varying separation distances *R*_*IJ*_. The resulting Φ_ML2_^(2)^(*R*_*IJ*_) predictions are shown as dashed lines for the two different solvents in Figure 2. Considering that the generated particle configurations for evaluating Φ_ML2_^(2)^(*R*_*IJ*_) are all different from those included in the original training data sets, the agreement with the curves obtained from the constrained MD simulations highlights the ability of the ML models to accurately interpolate between structures and smoothly predict the effective CG two-body potentials solely by learning the mean forces in FG simulations and not necessarily the scalar function itself.

### CG Many-Body Potential from ML Mean Forces

As mentioned above, when the effective CG potential of ligand-stabilized nanoparticles and other colloidal systems is described, typically two-body approximations are employed. However, one can expect many-body effects to be relevant for systems of nanoparticles in which the length scale of the “soft-deformable” layer of ligands, 2λ_s_, is of the order of the core size σ_c_ of the nanoparticles. More specifically, from simple geometrical arguments, one could expect three- and higher-body contributions to the effective CG potential Φ(**R**^*N*^) in eq 2 to be nonvanishing for size ratios *q* ≡ (2λ_s_)/σ_c_ > 0.14^44,45^ (see Figure 1). A common approach to deal with effective CG many-body potentials is to accurately determine the effective CG pair potential and treat the higher-body contributions as corrections.^7,27^ In particular, for a given configuration at fixed ***R***^*N*^ and in the absence of any external field, the αth component of the effective CG force on CG site *I* can be seen as the sum of an effective CG two-body (2) contribution (treated in a pairwise fashion) and effective CG three- and higher-body (3+) contributions, *F*_*I*,α_ = *F*_*I*,α_^(2)^+*F*_*I*,α_^(3+)^. The partitioning of the effective CG forces into individual two- and higher-order contributions is a convenient construction that has been typically adopted to disentangle the role of many-body interactions (for many technical reasons, however, mainly three-body interactions).^7,18,39^ A clear advantage of learning directly the individual mean forces as in our proposed scheme is that they already include such information as they are, by definition, gradients of the true effective CG many-body PMF.

**Figure 3:**
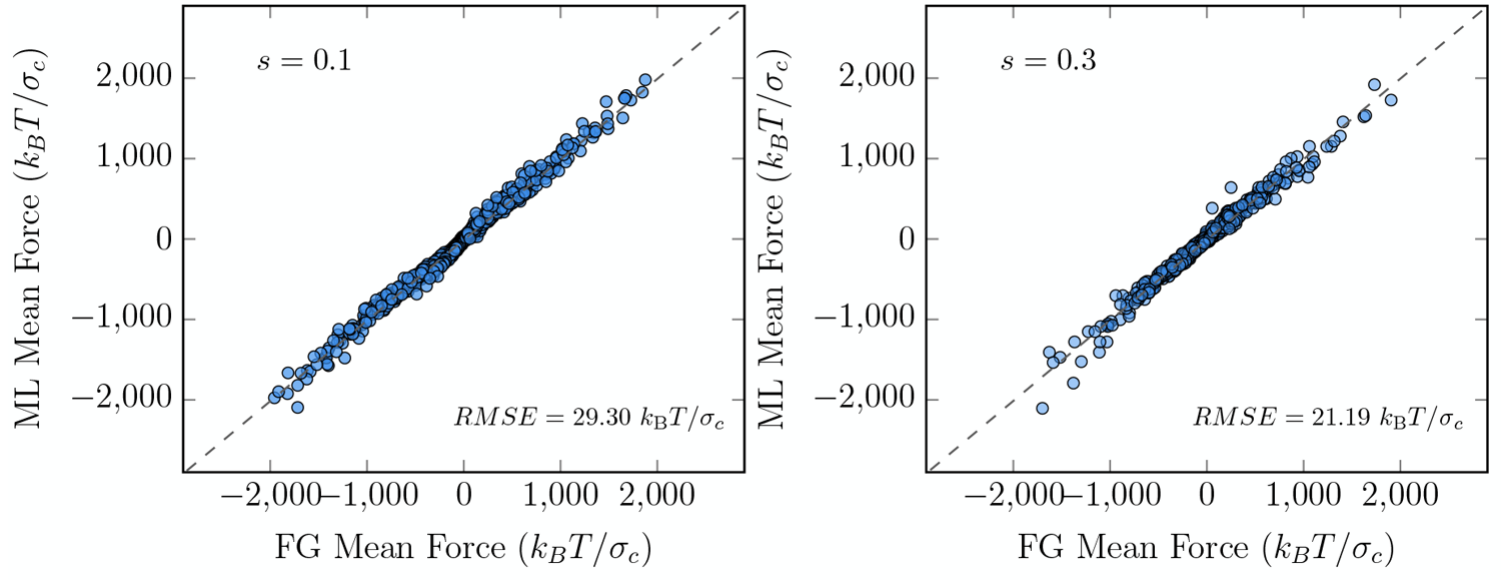
Parity plot comparing the vectorial components of the many-body mean forces (in *k*_B_*T*/σ_c_) on individual NPs in an implicit solvent with *s* = 0.1 (left) and *s* = 0.3 (right) predicted by the ML models with those calculated from the fine-grained (FG) models in systems consisting of low- and high-density clusters of 12 particles (see text for details).

Therefore, in order to demonstrate the generality of our ML approach in constructing effective CG potentials that incorporate many-body effects, we extend the method to larger systems composed of 12 nanoparticles. In this case, any higher-order body interaction (3+) should be captured in the effective CG ML potential Φ_ML12_ as the to-be learned mean forces are directly the gradients of the underlying true effective many-body PMF. For each solvent quality, a training data set is composed of a collection of configurations of 12 nanoparticles and the mean vectorial force components associated with each particle. Such configurations are obtained by first confining the 12 nanoparticles in a spherical domain using a repulsive wall. Subsequently, we fix the centers of mass of the nanoparticle cores and remove the walls. By varying the diameter of the confining sphere, we obtained high- and low-density configurations. Thus, the data set effectively contains configurations from very low density states, where barely two-body interactions are present, all the way to very high-density states, where the capping layers of different NPs simultaneously interact. After equilibration is reached in the MD simulations of the FG model, we select 100 samples at random and measure the mean forces on each nanoparticle core using constraint MD simulations. We again use eq 4 to fit the vectorial mean forces using simple linear regression of the gradients of the radial and angular SFs (see Descriptors: Symmetry Functions and their Gradients). The number of descriptors is *N*_SF_ = 123 and *N*_SF_ = 138 for *s* = 0.1 and *s* = 0.3, respectively. The resulting ML models present a correlation coefficient of *R*^2^ ≈ 0.998 and *R*^2^ ≈ 0.997, and RMSE values of 29.30 *k*_B_*T*/σ_c_ and 21.19 *k*_B_*T*/σ_c_, for a solvent with *s* = 0.1 and *s* = 0.3, respectively. Even with the simple linear model we use, the predicted effective CG forces obtained from our ML approach are in good agreement with those directly measured in MD simulations of the FG model, as shown in the parity plot in Figure 3 showing the many-body forces predicted by the ML models and those calculated from the fine-grained models.

**Figure 4:**
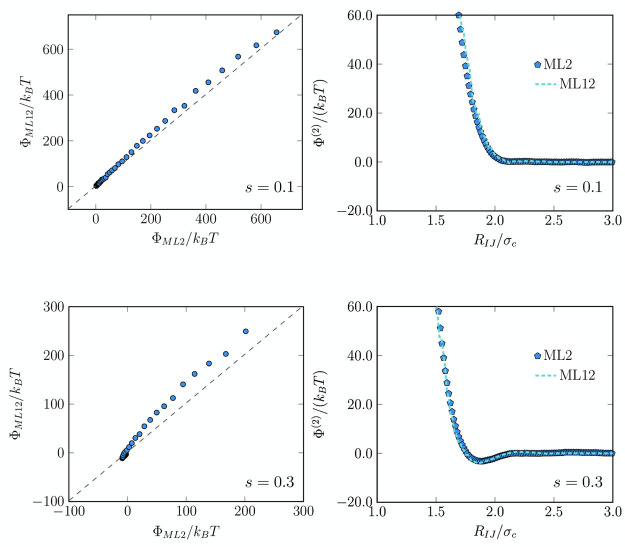
Left: Effective CG PMF values of systems composed of 12 NPs in an implicit solvent with *s* = 0.1 (top-left) and *s* = 0.3 (bottom-left) as predicted by the ML models Φ_ML2_(*R*_*IJ*_) and Φ_ML12_(**R**^12^) (see the text for details). Right: Effective interaction between two particles as a function of the interparticle separation distance *R*_*IJ*_ as obtained from the ML Φ_ML2_(*R*_*IJ*_) model (filled pentagons) and as obtained from the Φ_ML12_(*R*_*IJ*_) model (dashed lines) for NPs in solvents with *s* = 0.1 (top-right) and *s* = 0.3 (bottom-right).

As a natural test of the effective CG many-body potential of mean force Φ_ML12_ as obtained from eq 8 using the 12 NP system, we evaluate the effective CG interaction energy between only two particles Φ_ML12_^(2)^(*R*_*IJ*_) as a function of their separation distance *R*_*IJ*_ and compare it against the one obtained for the strictly two-body system Φ_ML2_^(2)^(*R*_*IJ*_) as described above, which accurately described the effective CG PMF directly obtained from constrained MD simulations of the FG model as shown in Figure 2. The results are shown in the right panels of Figure 4, where we can appreciate the excellent match between the effective CG potentials for a solvent with *s* = 0.1 and *s* = 0.3. We emphasize that no shifting constants have been used to make the Φ_ML12_^(2)^(*R*_*IJ*_) and Φ_ML2_^(2)^(*R*_*IJ*_) curves coincide. The observed agreement demonstrates not only that the *R*_*IJ*_-dependence of the effective CG two-body interaction potential is well captured but that since the model is constructed on the basis of individual particle contributions it is able to accurately differentiate between local configurations.

The values of the effective CG PMF Φ_ML12_(**R**^12^) for different clusters of 12 particles as obtained by summing the two-body effective potential Φ_ML2_(*R*_*IJ*_) between pairs of particles and as predicted by Φ_ML12_(**R**^12^) are compared in the left panels of Figure 4. We can appreciate that, while both descriptions strongly correlate, the Φ_ML12_(**R**^12^) potential tends to overestimate the strength of the interactions, as the values are systematically higher (more positive) than those predicted by the Φ_ML2_(*R*_*IJ*_) potential.

**Figure 5:**
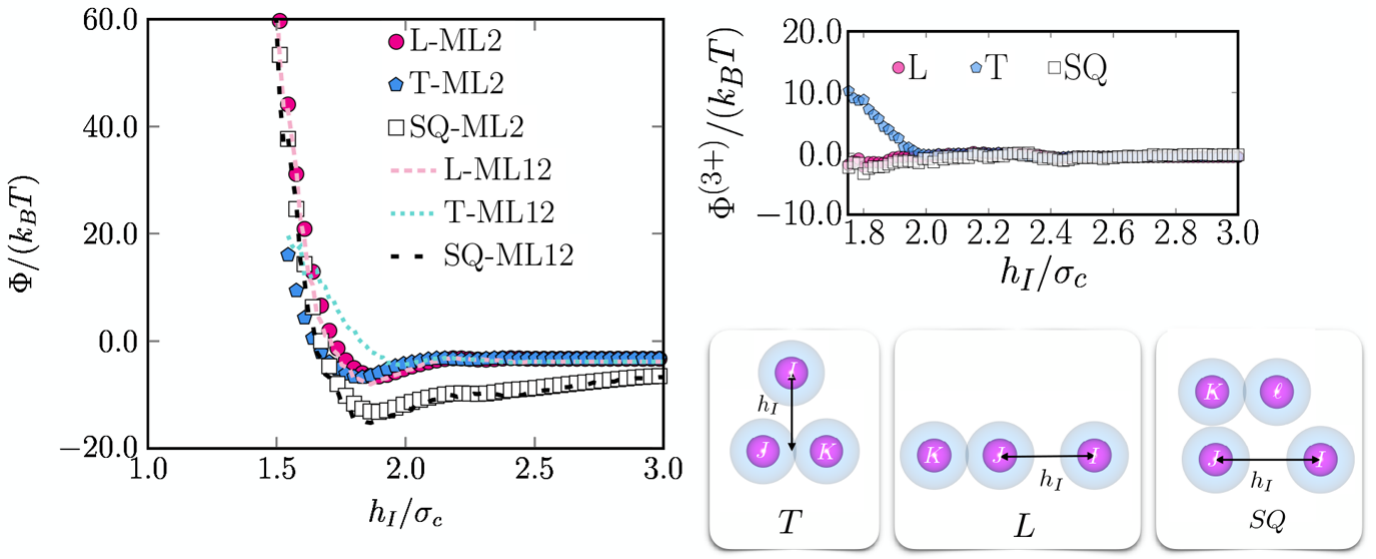
Left: The effective CG potential of mean force Φ as a function of position *h*_*I*_ of particle *I* relative to the position of the remaining fixed particles as shown in the pictorial sketches. Values are shown for a triplet of particles in bad solvent (*s* = 0.3) ordered in triangular (T) and linear (L) configurations as well as for a quartet (SQ) (see right bottom panel). Filled symbols correspond to the potential values obtained from the two-body approximation (Φ_ML2_), whereas the dashed lines represent those obtained from the ML potential Φ_ML12_ constructed from the linear fit of the mean forces in systems containing clusters of 12 particles. Right: Difference between the effective CG many- and two-body PMFs in the considered configurations, Φ^(3+)^. Note that curves of Φ on the left are obtained by keeping particles *J*, *K*, and  fixed at a separation distance  = 1.88σ_c_.

To better appreciate the effect of many-body effects on the effective CG potential, we focus on the effective three-body correction in a solvent with *s* = 0.3. To this end, we evaluate the effective CG PMF in systems containing 3 particles in triangular (T) and linear (L) configurations using the ML CG potential constructed with only two-body terms Φ_ML2_ and the one containing the many-body corrections Φ_ML12_ from the ML fits of the mean forces. To generate the configurations, we fix two particles at a separation distance *R*_*JK*_/σ_c_ = 1.88 and place the third particle *I* at different positions *h*_*I*_ relative to the two fixed particles (see the pictorial sketches in Figure 5). The resulting curves are shown in Figure 5. As observed, the curves of Φ_ML2_ and Φ_ML12_ naturally take the value of Φ^(2)^(1.88σ_c_) ≈ −3.3 *k*_B_*T* at large separation distances, as the effect of the third particle becomes negligible. For the L configurations, we notice that the Φ_ML2_ and Φ_ML12_ curves exactly overlap at all *h*_*I*_ distances. This is because particles *I* and *K* do not effectively interact at any *h*_*I*_, thus ruling out any three-body interactions. However, one can immediately appreciate that, in the T configurations, the three-body contribution is nonvanishing as the Φ_ML2_ and Φ_ML12_ curves slightly differ from each other. In particular, we find that the ML effective CG PMF based on two-body contributions Φ_ML2_ overestimates the effective CG attractive (*cohesive*) interactions, which is in agreement with the results above. Furthermore, the steric repulsion between a triplet of particles at short distances becomes slightly stronger if the three-body correction is considered. This positive (repulsive) contribution to the CG PMF incorporated in Φ^(3+)^ appears to be common to systems exhibiting an effective CG two-body attractive interaction (e.g., colloid–polymer mixtures^26^). Finally, we also show in Figure 5 the effective CG PMF for a system of four particles as illustrated in the inset SQ. As expected for such a configuration, the effective CG many-body effects are negligible, but it clearly shows that the model is able to reproduce the correct energies. This also holds for clusters with more particles as we have discussed above.

### Testing the ML Models: Phase Behavior and Structure

**Figure 6:**
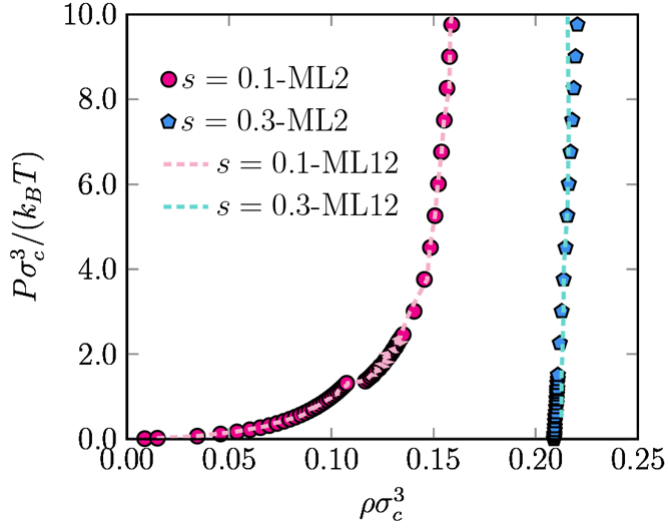
Equation of state, reduced pressure *Pσ*_c_^3^/*k*_B_*T* as a function of reduced density *ρσ*_c_^3^, for a good solvent *s* = 0.1 and a bad solvent *s* = 0.3 as obtained by MC simulations using the effective CG two-body Φ_ML2_ potential (filled symbols) and those obtained using the effective CG many-body Φ_ML12_ potential (dashed lines).

To investigate the effect of nonvanishing many-body corrections on the effective CG many-body PMF, we focus on the phase behavior of NPs in two different solvents. More precisely, we compute the equation of state (EOS) of 3D bulk systems consisting of NPs in both solvents by performing isothermal–isobaric (*NPT*) MC simulations using the effective CG two-body Φ_ML2_ and the effective CG many-body Φ_ML12_ potentials of mean force. We perform simulations on a system of *N* = 500 nanoparticles and obtain the equilibrium states by either compression from the low-density fluid phase or expansion from a high-density face-centered-cubic (FCC) phase. In Figure 6, we plot the equations of state, reduced pressure *Pσ*_c_^3^/*k*_B_*T* as a function of reduced density *ρσ*_c_^3^, for both solvents. Interestingly, in a good solvent (*s* = 0.1), both the effective CG Φ_ML2_ and Φ_ML12_ potentials yield nearly identical curves. We notice that under such solvent conditions, the system exhibits a low-density fluid phase for *ρσ*_c_^3^ < 0.10 that eventually undergoes a first-order phase transition to a FCC crystal with a density *ρσ*_c_^3^ ≈ 0.11. The reasonable match between the two effective CG potentials, which was also observed in the parity plot of Figure 4 leads us to conclude that, for *s* = 0.1, the effective CG two-body approximation is indeed reasonable and that the many-body corrections do not alter the phase behavior of the system. For a solvent quality *s* = 0.3, we observe a FCC crystal at a density of *ρσ*_c_^3^ ≈ 0.21 using both the effective CG Φ_ML2_ and Φ_ML12_ potentials. This is due to the bad solvent conditions, which induce a strong effective attraction between the nanoparticles. Indeed, even if we start the simulations from very low-density configurations at very low pressures, we observe the formation of clusters that eventually nucleate into an FCC phase. We have attempted to obtain the density of the coexisting fluid phase by direct-coexistence simulations, where we start with an equilibrated FCC crystal slab in the center of an elongated box in contact with a vacuum; however, we do not detect any particles leaving the crystal to migrate to the gas phase in our *NVT* simulations. The total effective energy for a triplet of particles in a bad solvent (Figure 5) indicates that the linear configuration is energetically more favorable than the triangular one. In systems of NPs interacting with a short-range square-well potential grafted by up to 12 fully flexible chains consisting of up to 14 hard beads^46^ as well as in moderately polymer-grafted NPs in a polymer melt,^47^ this type of effective interaction may lead to the formation of anisotropic structures like stripes and disks. Nevertheless, in our simulations with *N* = 500 particles in bad solvent, the small clusters rapidly aggregate into the equilibrium 3D FCC phase, which indicates that the effective two-body interactions outweigh the repulsive contributions. We therefore conclude that the system exhibits a very broad phase coexistence between an infinitely dilute gas phase and an FCC phase with a coexisting density *ρσ*_c_^3^ ≈ 0.21 for a solvent quality *s* = 0.3. Additionally, we note that there are small deviations in the equations of state as obtained by using the effective CG Φ_ML2_ and Φ_ML12_ potentials, signaling the importance of the many-body effects on the effective CG potential. More specifically, we observe that the pressure is underestimated using the effective CG Φ_ML2_ in comparison with the Φ_ML12_ potential at high densities, which is in agreement with the parity plot of Figure 4, showing that the repulsion is underestimated by the effective CG Φ_ML2_ PMF.

**Figure 7:**
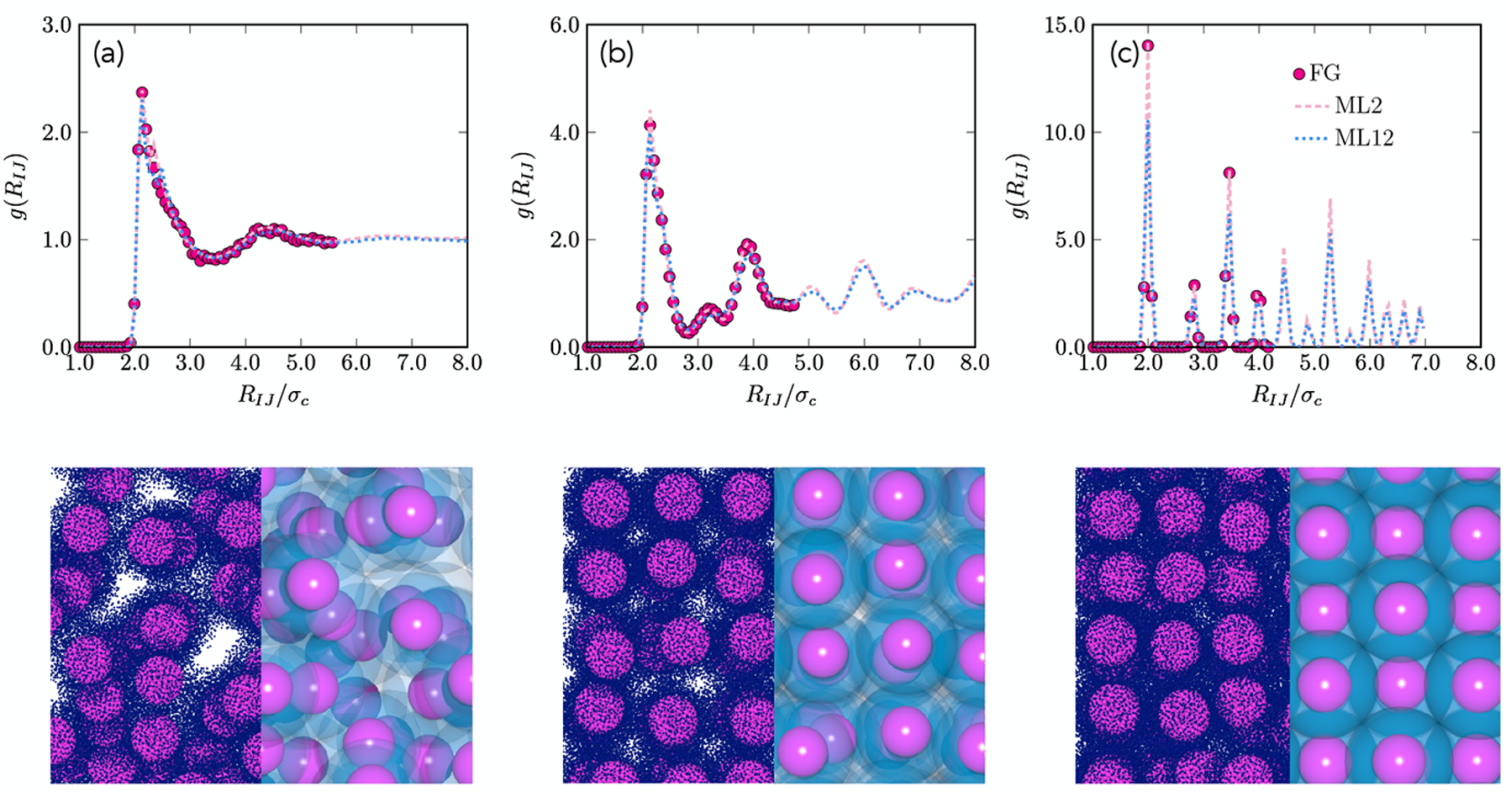
Radial distribution functions *g*(*R*_*IJ*_) as a function of the distance *R*_*IJ*_ between the nanoparticle cores for a solvent with *s* = 0.1 as obtained from MD simulations of the FG model (filled circles) and from MC simulations using the effective CG two-body Φ_ML2_ potential (dashed line) and many-body Φ_ML12_ potential (dotted line) at density *ρσ*_c_^3^: (a) 0.07, (b) 0.12, and (c) 0.18. The corresponding typical configurations obtained from MD simulations of the FG and from MC simulations of the CG model are shown in the panels below.

Finally, to further validate the effective CG many-body potentials, we compute the radial distribution functions *g*(*R*_*IJ*_) as a function of the distance *R*_*IJ*_ between the nanoparticle cores in the equilibrium phases of the NPs using MC simulations and compare them against results obtained from extensive *NVT* unconstrained MD simulations of the FG model. In particular, we focus on a system with solvent quality *s* = 0.1 as it exhibits a low-density fluid phase and a *soft* FCC crystal that, in our simulations, can be continuously compressed from *ρσ*_c_^3^ ≈ 0.11 all the way to *ρσ*_c_^3^ ≈ 0.18 preserving the same symmetry but just changing the lattice constant. The *g*(*R*_*IJ*_)’s as measured from MC simulations using the machine-learned effective CG many-body potentials Φ_ML2_ and Φ_ML12_ along with the *g*(*R*_*IJ*_)’s obtained from MD simulations of the FG model are shown in Figure 7 for varying densities *ρσ*_c_^3^ = 0.07, 0.12, and 0.18. We clearly observe that the structures of the phases obtained from both CG models; i.e., the effective CG Φ_ML2_ and Φ_ML12_ potentials of mean force match very accurately those of the FG model. These results evidence the ability of the proposed ML method in constructing the effective CG many-body potentials by learning solely the mean forces sampled in the FG model.

## Conclusions

In summary, we have introduced a machine-learning approach to construct effective coarse-grained many-body potentials for complex ligand-stabilized nanoparticles by representing the mean forces sampled from constrained simulations of a fine-grained model in terms of gradients of structural descriptors. For the specific model of NPs that we have studied in an implicit solvent of varying quality, the effective coarse-grained two-body contribution Φ^(2)^ to the PMF in the presence of a bad solvent is a nonmonotonic function of the separation distance between two particles and exhibits a deep minimum, corresponding to an effective coarse-grained attractive interaction. In contrast, in a good solvent, the effective coarse-grained two-body PMF is fully repulsive. As judged from the results of MC simulations using the effective coarse-grained two-body potential of mean force Φ_ML2_ and the effective many-body potential of mean force Φ_ML12_, we find that both CG models reproduce accurately the phase behavior and the structure of the fluid and crystal phases of the FG model as they match the equation of state and the radial distribution functions at varying thermodynamic state points. In summary, our simulations indicate that for nanoparticles stabilized by ligands with a commonly used chain length, the impact of many-body effects on phase behavior is minimal, except for high-density crystal phases. This is a significant and nontrivial finding.

The multiscale methodology presented in this work constitutes a general bottom-up coarse-graining strategy where the mean forces acting on CG sites, which are extracted from reference FG simulations, are represented using a simple linear model in terms of gradients of Behler and Parrinello symmetry functions. The linearity of the model allows one to define a simple function representing scalar effective CG many-body potentials of mean force as a function of all CG-site coordinates. This allows us to bypass the prior identification of suitable reaction coordinates (collective variables) to measure gradients and subsequently integrate them to obtain a scalar many-body potential of mean force as is required in the methodology of ref (22) and in our previous work in ref (27).

**Figure 8:**
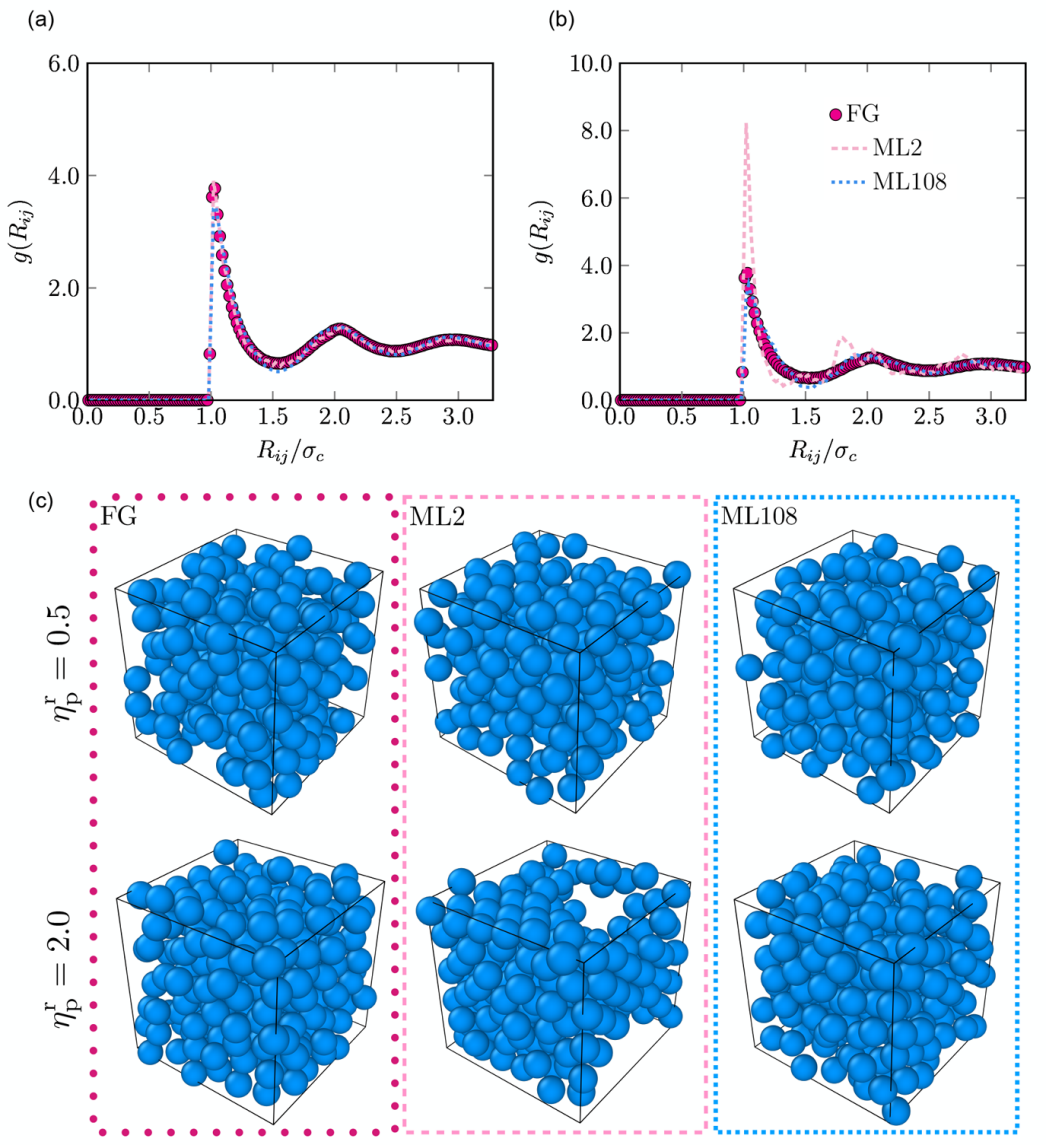
Colloid-colloid pair correlation function measured in bulk systems at colloid packing fraction η_c_ = 0.45 and (a) reservoir polymer packing fraction η_p_^r^ = 0.5 and (b) η_p_^r^ = 2.0. Filled circles correspond to the distributions measured in the FG model, while the dashed pink and dotted blue lines represent the results obtained with the CG ML2 and ML108 potentials, respectively. (c) Typical configurations of the simulated systems. See Supporting Information for more details.

Finally, we stress that although we have illustrated the method by applying it to an FG model of ligand-stabilized NPs, the framework is generic and can be extended to other relevant systems. In Figure 8 and in the Supporting Information, we present an application to a system of colloid–polymer mixtures, where coarse-grained many-body interactions are pronounced. We employ the so-called pseudo Asakura Oosawa model, where the colloids are represented by hard spheres, the pseudo colloid–polymer interactions are also treated by a pseudo hard-sphere-like potential, and the polymers are ideal. The diameter σ_p_ of the polymer-coils is set equal to the diameter of the colloids σ_c_. We show in Figure 8 and in the Supporting Information that a coarse-grained description of such a system based purely on effective pairwise depletion interactions, ML2, leads to strong deviations from the FG model. In contrast, the FG model can be accurately represented by the effective CG potential, ML108, that accounts for many-body contributions.

The current method, which relies on descriptors of local environments that are spherically symmetric, has demonstrated promising results for systems with isotropic interactions. In principle, our method could be extended to systems in which the interactions are anisotropic, such as faceted nanocrystal cores or particles with inhomogeneous distributions of ligands on their surfaces. However, this would require descriptors that can encode information about the orientational dependence of the anisotropic interactions, which we will explore in future work. Our simple yet accurate force-matching coarse-graining framework will enable accurate and fast simulations of the effective many-body systems to characterize, understand, and predict the phase behavior and structure of relevant soft-matter systems such as suspensions of charged colloids or microgel particles.

## Methods

### Model and Simulation Details

#### Implicit Solvent

To mimic the effect of a solvent in an implicit fashion, we follow the approach by Fan et al.,^34^ where the pair interactions between nonbonded beads in the nanoparticles are modeled through a modified LJ potential:
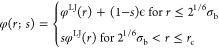
11where
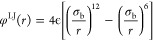
12is the standard LJ potential, σ_b_ = 0.47 nm and ϵ = 0.8365 kcal mol^–1^ are the length- and energy-scale parameters of the pair interactions, respectively, *r* is the separation distance between pairs of beads, and *r*_c_ = 1.2 nm is the cutoff radius of the interaction. The parameter *s* defines the strength of the attractive LJ interactions relative to the original MARTINI value. In order to mimic good- and poor-solvent conditions for the ligand chains, we use two values of *s*, namely, *s* = 0.1 and *s* = 0.3. Note that the limiting case of *s* = 1 recovers the original LJ potential and mimics an extremely bad solvent (or vacuum) for the NPs. Intramolecular interactions within the chains, acting on the centers of bonded beads, are described via a harmonic bond-stretching potential:
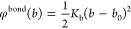
13where *K*_b_ = 149.3787 kcal mol^–1^ nm^–2^ is the bond force constant, and *b* and *b*_0_ = 0.47 nm the instantaneous and equilibrium bond distances, respectively. Similarly, the angle-bending between three connected beads is modeled using a harmonic potential,
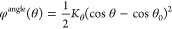
14where *K*_θ_ = 2.9876 kcal mol^–1^ is the angle force constant, and θ and θ_0_ = 180° are the instantaneous and equilibrium angle-bending values, respectively. Interactions between NC cores are neglected, as these forces, for small NCs, are typically much weaker than the ligand–ligand interactions.^48,49^

#### Molecular Dynamics Simulations of the FG Model

All molecular dynamics (MD) simulations on systems with different number of NPs are performed using the software package LAMMPS.^50^ The simulation box is typically a cubic cell of volume *V* where periodic boundary conditions are applied in all directions. Prior to MD simulations, overlaps between ligand beads in the initial configurations are removed by energy minimization using the steepest-descent algorithm. Simulations are then performed in the canonical (*NVT*) ensemble at a temperature *T* = 300 K, which is kept constant via a Nosé–Hoover thermostat. The equations of motion are integrated with a typical MARTINI time step of *δt* = 20 fs, where the NP cores can be either “diffusing” as rigid bodies or have their centers of mass spatially constrained within the simulation box volume. Typically, constraint MD simulations for computing the mean forces are run for up to 5 × 10^7^ steps, where statistics are collected over the last 3 × 10^6^ steps.

For validation of the CG potentials obtained by direct fitting of the mean forces, we perform extra MD simulations of the FG model at different densities by using *N* = 108 particles. These simulations are run for up to 1 × 10^8^ steps.

### Thermodynamic Consistent Coarse-Graining

The effective coarse-grained potential of mean force Φ(***R***^*N*^) described in the main text can alternatively be formally conceived in the context of the *effective one-component Hamiltonian* formalism.^45,51^ To demonstrate this, we define the total Hamiltonian as a sum of interaction Hamiltonians describing the interactions between the *N* nanoparticle cores , the interactions between the *N* nanoparticle cores and the *n*_*l*_ ligand beads , and the interactions between the *n*_*l*_ ligand beads 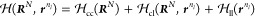
15where ***R***^*N*^ denote the coordinates of the nanoparticle cores, which are treated as rigid bodies consisting of *n*_c_ beads, and ***r***^*n*_*l*_^ the coordinates of the ligand beads. In the canonical ensemble (*N*, *n*_*l*_, *V*, *T*), the partition function, after carrying out the trivial integrations over the momenta reads
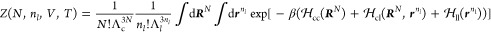
16where Λ_α_ denotes the *thermal de Broglie wavelength* of species α. By integrating out the degrees of freedom of the ligand beads, we obtain
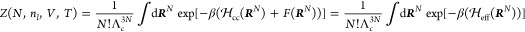
17where we have mapped the fine-grained system of *N* nanoparticle cores and *n*_l_ ligand beads described by an interaction Hamiltonian  onto an effective coarse-grained system of nanoparticles that is described by an effective one-component Hamiltonian . Here, we have defined the Helmholtz free energy of the ligand beads in the external field of a fixed configuration of nanoparticle cores at coordinates ***R***^*N*^
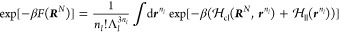
18

From these definitions, it follows that in the case that an observable  only depends on the coordinates of the centers of mass of the nanoparticles ***R***^*N*^, the ensemble average  is identical in the full system and in the effective one-component system
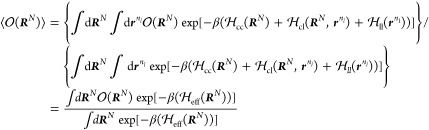
19Hence, the mean force acting on the center of mass of nanoparticle *I* at a fixed configuration ***R***^*N*^ can be computed as
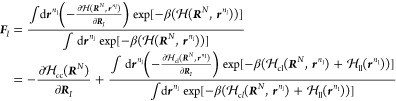
20where the first term  is the force due to the bare interactions between the nanoparticle cores, and the last term is the force of the ligand beads on nanoparticle *I* averaged over all possible configurations of the ligand beads.

### Fitting Procedure

#### Training Data Set

As detailed in the main text, two different data sets are built: (i) one containing information about a pair of particles at different separation distances and (ii) one for configurations of 12 particles at varying density. In (i), the mean forces acting on the center of mass of the nanoparticle cores are collected over a total of 250 separation distances. Hence, a total of 2 × 3 × 250 examples are used to construct the model. In case (ii), the mean forces on all the particles are measured in 100 constraint MD simulations, resulting in a total number of 12 × 3 × 100 examples.

#### Descriptors: Symmetry Functions and their Gradients

To describe the local environment of a particle, we employ the SFs proposed by Behler and Parrinello^43^ to represent high-dimensional potential-energy surfaces based on neural networks. More precisely, to characterize the local environment of a particle within a cutoff distance *R*_c_ we employ the particle-centered spherically symmetric radial SFs, defined as
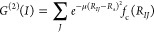
21which is a sum of Gaussians, where the parameters μ and *R*_s_ control the width and position of the Gaussians with respect to particle *I*, respectively, and where *f*_c_(*R*_*IJ*_) is a cutoff function which decays monotonically and smoothly goes to 0 in both value and slope at the cutoff distance *R*_c_
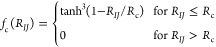
22

The second type of SF is the angular SF, *G*^(3)^, which contains information on the angular correlations and is defined as
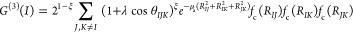
23where the indices *J* and *K* run over all the neighbors of particle *I*, and ξ and λ are two parameters that determine the shape of the function. The parameter λ can have values of +1 or −1 and determines the angle θ_*IJK*_ at which the angular part of the function has its maximum. The parameters ξ and μ_a_ control the angular and radial resolution, respectively.

The gradients of the radial SF, which we directly use to represent the vectorial forces, can be straightforwardly calculated by the repeated use of the chain rule for derivation. The gradient does take a different form if the gradient is taken with respect to the particle for which the SF is computed ∇_*I*_*G*^(2)^(*I*) or with respect to a different neighboring particle ∇_*J*_*G*^(2)^(*I*), where *J* ≠ *I*. These analytical and continuous gradients are given by
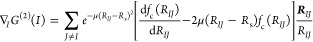
24and
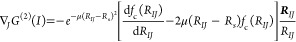
25Note that we use the convention that ***R***_*IJ*_ = ***R***_*I*_ – ***R***_*J*_ and *R*_*IJ*_ = |***R***_*IJ*_|. For the angular SF, computation of the gradient is still a straightforward application of the chain rule, although it is more involved than for the radial SF. In this case, the gradients read
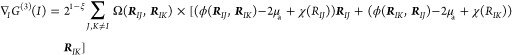
26and
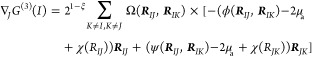
27

where we have used the following auxiliary functions as introduced in ref (52):
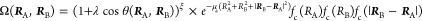
28
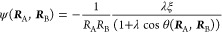
29
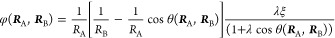
30
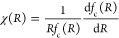
31

#### Selection of Descriptors

The feature selection method employed in this work is described in detail in refs (25 and 26). Here, we present only a brief summary. We will use the acronym SFs to refer to the symmetry functions and their gradients interchangeably. The first step of the approach is to create a large but manageable pool of candidate descriptors (gradients of SFs). Such a procedure is accomplished by calculating, for every particle in the data set, *M* SFs with varying parameter values, which in turn are selected following simple heuristic rules with the goal of capturing most of the many-body correlations within a certain cutoff radius. In all the cases presented in this work, we have used a cutoff value of *R*_c_ = 2.5σ_c_. The parameters for the radial SFs employed for the generation of the initial pool for constructing the models of systems containing two nanoparticles for solvents *s* = 0.1, 0.3 are μ/σ_c_^–2^={0.00001, 0.0001, 0.001, 1.715, 3.429, 5.144, 6.858, 8.572, 10.286, 12.000, 13.715, 15.429, 17.143, 18.857, 20.572, 22.286, 24.000, 26.000, 28.000, 30.000, 32.000, 34.0000} and *R*_s_/σ_c_ = {0.0, 0.1, 0.2, 0.3, 0.4, 0.5, 0.6, 0.7, 0.8, 0.9, 1.0, 1.1, 1.2, 1.3, 1.4}. The ones for the radial and angular SFs used in the models for systems of 12 nanoparticles in both solvents are μ/σ_c_^–2^ = {0.001, 1.715, 3.429, 5.144, 6.858, 8.572, 10.286, 12.000, 13.715, 15.429}, *R*_s_/σ_c_ = {0.0, 0.1, 0.2, 0.3, 0.4, 0.5, 0.6, 0.7, 0.8, 0.9}, μ_a_/σ_c_^–2^ = {0.0001, 0.001, 0.01, 0.1, 1.0, 2.0, 4.0, 8.0}, λ = {1, – 1}, and ξ = {1.0, 4.0, 8.0, 12.0}.

In a second step, an optimal subset of *N*_SF_ < *M* SFs is selected from the pool in a stepwise fashion. The optimal subset should capture the most relevant features of the local environment of a particle, as it is subsequently used as the basis of a regression scheme to approximate the mean force of a particle. The first selected SF corresponds to the one with the largest correlation with the target function as quantified by the square of the Pearson correlation coefficient, defined as
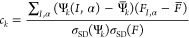
32where  represents the sum of the derivatives of the *k*th SF for all *N* particles with respect to particle *I*, as used in the definition of the model in eq 7. *F*_*I*,α_ denotes the target mean force component on particle *I*.  and F̅ correspond to arithmetic means over all the configurations in the data set, and σ_SD_(Ψ_*k*_) and σ_SD_(*F*) to their standard deviations. Note that summation over vectorial components is implied above. The second SF is then selected to be the one that maximizes the increase in the linear correlation between the set of selected SFs and the target data as determined by the coefficient of multiple correlations, whose square is given by
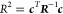
33where **c**^*T*^ = (*c*_1_, *c*_2_, ...) is the vector whose *i*th component is given by the Pearson correlation coefficient, *c*_*i*_, between the *i*th SF (gradient) and the target data, and **R** is the correlation matrix of the current set of SFs with elements **R**_*ij*_ representing the Pearson correlation function between the *i*th and *j*th SF. In the case of only one SF, *R*^2^ reduces to *c*_*i*_^2^. *R*^2^ can also be computed as the fraction of variance that is explained by a linear fit (including an intercept) of the target function in terms of the SFs in the set. The latter method of computing *R*^2^ is slightly more expensive but is numerically more stable.^25^ By maximizing the increase in the linear correlation with the target variable, we guarantee that only SFs that add relevant information are selected, while SFs that poorly correlate with the particle forces or free energy are avoided along with highly correlated SFs which add redundant information.^25^ This process is repeated iteratively, and further SFs are selected until *R*^2^ stops increasing appreciably. This indicates that the remaining SFs in the pool add negligible (irrelevant) information to the model. Such a procedure constitutes a simple rule to optimize the number of selected SFs, which are then used to approximate the target function via simple linear regression.

## Supporting Information Available

Additional details of the colloid–polymer mixture system (PDF)
