# Supplementary material for: Coarse-Grained Many-Body Potentials of Ligand-Stabilized Nanoparticles from Machine-Learned Mean Forces
Source: ACS Nano. 2023 Nov 27;17(23):23391–404. doi: 10.1021/acsnano.3c04162 (PMC10722599; doi:10.1021/acsnano.3c04162)
Supplement: Supplementary file 1 — nn3c04162_si_001.pdf [file nn3c04162_si_001.pdf]

Supporting Information
Coarse-grained many-body potentials of ligand-stabilized nanoparticles from machine-learned mean forces

Giuliana Giunta,[*,†,‡] Gerardo Campos-Villalobos,[†] and Marjolein Dijkstra[*,†]

†*Soft Condensed Matter, Debye Institute for Nanomaterials Science, Utrecht University, Princetonplein 5, 3584 CC Utrecht, The Netherlands*

‡*Current address: BASF SE, Carl-Bosch-Strasse 38, 67056 Ludwigshafen am Rhein, Germany*

E-mail: giuliana.giunta@basf.com; m.dijkstra@uu.nl

Colloid-Polymer Mixtures

To demonstrate the robustness of our coarse-graining method, we herein apply it to a model suspension of sterically-stabilized colloidal particles and non-adsorbing ideal polymers for which a wealth of data exists on the phase behavior and structure.[1–4] Departing from the mean forces measured on the colloidal particles in the full binary system, we construct an effective one-component (colloids-only) potential that accurately incorporates the polymer-mediated many-body interactions.

Fine-Grained Model

In the original Asakura-Oosawa (AO) model for colloid-polymer mixtures,[5–7] colloidal particles are represented as hard spheres of diameter σ_{c} whereas the polymer coils with diameter σ_{p} are treated as ideal point particles as regards their mutual interactions. The colloid-polymer pair interaction is hard-sphere-like such that their distance of closest approach is $\sigma_{\mathrm{cp}} = (\sigma_{\mathrm{c}} + \sigma_{\mathrm{p}})/2$. Here, we employ an adapted AO model based on continuous pair potentials, which is fully suitable for Molecular Dynamics (MD) simulations. We will refer to this model as the pseudo Asakura-Oosawa (PAO) model. In particular, we consider a system consisting of N_{c} colloidal spheres at coordinates $\{\boldsymbol{R}_i\}$ with $i = 1, \ldots, N_{\mathrm{c}}$, and N_{p} polymer coils at positions $\{\boldsymbol{r}_j\}$ with $j = 1, \ldots, N_{\mathrm{p}}$ with a size ratio $q = \sigma_{\mathrm{p}}/\sigma_{\mathrm{c}}$ in a volume V at temperature T. The hard-sphere-like pair interactions in the pseudo-AO model are represented by a cut-and-shifted Mie potential:

$$\phi_{\mathrm{cc}}(R_{ij}) = \begin{cases} \frac{\lambda_{\mathrm{r}}}{\lambda_{\mathrm{r}}-\lambda_{\mathrm{a}}} \left(\frac{\lambda_{\mathrm{r}}}{\lambda_{\mathrm{a}}}\right)^{\frac{\lambda_{\mathrm{a}}}{\lambda_{\mathrm{r}}-\lambda_{\mathrm{a}}}} \epsilon \left[\left(\frac{\sigma_{\mathrm{c}}}{R_{ij}}\right)^{\lambda_{\mathrm{r}}} - \left(\frac{\sigma_{\mathrm{c}}}{R_{ij}}\right)^{\lambda_{\mathrm{a}}} \right] + \epsilon & \text{for } R_{ij} < \sigma_{\mathrm{c}} \left(\frac{\lambda_{\mathrm{r}}}{\lambda_{\mathrm{a}}}\right)^{\frac{1}{\lambda_{\mathrm{r}}-\lambda_{\mathrm{a}}}} \\ 0 & \text{otherwise ,} \end{cases} \tag{1}$$

$$\phi_{\rm cp}(|\boldsymbol{R}_i - \boldsymbol{r}_j|) = \begin{cases} \frac{\lambda_{\rm r}}{\lambda_{\rm r}-\lambda_{\rm a}} \left(\frac{\lambda_{\rm r}}{\lambda_{\rm a}}\right)^{\frac{\lambda_{\rm a}}{\lambda_{\rm r}-\lambda_{\rm a}}} \epsilon \left[\left(\frac{\sigma_{\rm cp}}{|\boldsymbol{R}_i-\boldsymbol{r}_j|}\right)^{\lambda_{\rm r}} - \left(\frac{\sigma_{\rm cp}}{|\boldsymbol{R}_i-\boldsymbol{r}_j|}\right)^{\lambda_{\rm a}} \right] + \epsilon & \text{for } |\boldsymbol{R}_i - \boldsymbol{r}_j| < \sigma_{\rm cp} \left(\frac{\lambda_{\rm r}}{\lambda_{\rm a}}\right)^{\frac{1}{\lambda_{\rm r}-\lambda_{\rm a}}} \\ 0 & \text{otherwise ,} \end{cases} \tag{2}$$

with $R_{ij} = |\boldsymbol{R}_i - \boldsymbol{R}_j|$ the center-of-mass distance between colloid i and j, ϵ and $\sigma_{\rm c}$, $\sigma_{\rm cp}$ denote the energy and length scales of the interaction, respectively. By setting $\lambda_{\rm r} = 50$ and $\lambda_{\rm a} = 49$ and considering a reduced temperature of $k_BT/\epsilon = 1.5$ (k_B being the Boltzmann constant), the pair potential reproduces the volumetric, structural, and dynamic properties of the discontinuous hard-sphere potential over the entire fluid range.[8]

The ideal character of the polymers is attained by neglecting their mutual pair interactions during the MD simulations, i.e., $\phi_{\rm pp}(r_{ij}) = 0$. We achieve this in LAMMPS[9] using the `neigh_modify exclude` command.

Coarse-Grained Models

For the purpose of mapping the original AO binary system onto a coarse-grained one by formally integrating out the degrees of freedom of the polymer coils, one typically treats the polymer coils grand-canonically, where the fugacity of the polymers $z_{\rm p}$, or equivalently the polymer reservoir packing fraction $\eta_{\rm p}^{\rm r} \equiv \pi\sigma_{\rm p}^3 z_{\rm p}/6$, is fixed. By departing from the thermodynamic potential $F(N_{\rm c}, z_{\rm p}, V, T)$ of the binary system, it can be demonstrated that the original AO binary mixture can be described by an effective colloids-only Hamiltonian $\mathcal{H}_{\rm eff} = \mathcal{H}_{\rm cc} + \Omega$, where Ω denotes the grand potential of a "sea" of ideal polymers at fugacity $z_{\rm p}$ in the external field of a fixed configuration of $N_{\rm c}$ colloids.[1,3] For the original AO model, the grand potential Ω is simply the negative of the free volume available for the polymer in the fixed configuration of $N_{\rm c}$ colloids weighted by the polymer fugacity in the reservoir, i.e., $\Omega = -z_{\rm p} V_{\rm f}(\{\boldsymbol{R}_i\})$. From the definition of the grand potential, one can immediately recognize the many-body character of the effective one-component coarse-grained model, where the free volume, and therefore the size ratio q, plays a crucial role. In fact, for $q < 0.1547$, the higher-order

contributions are zero for the original AO model, and hence a mapping onto an effective one-component system with an effective Hamiltonian based on pairwise additive depletion potentials is exact.[1,10] For larger q, the model based solely on the pairwise approximation exhibits a phase behaviour that strongly deviates from that observed in the system where three- and higher-body interactions are considered.

In order to coarse-grain the PAO model with a size ratio $q = 1.0$ using our multiscale approach, we rely on simulations of the binary system in the canonical $(N_{\mathrm{c}}, N_{\mathrm{p}}, V, T)$ ensemble. However, to create a model that is thermodynamically consistent and transferable among different state points, the amount of polymer in the suspension is set in a quasi-grand-canonical fashion. More specifically, we follow the results from Free Volume Theory (FVT)[11] where a simple relationship exists between the equilibrium packing fraction of polymer in the reservoir ($\eta_{\mathrm{p}}^{\mathrm{r}}$) and that in the suspension containing the colloids (η_{p})

$$\eta_{\mathrm{p}}^{\mathrm{r}} = \frac{\eta_{\mathrm{p}}}{\alpha_{\mathrm{FVT}}}, \tag{3}$$

where the parameter α_{FVT} depends on the colloid packing fraction η_{c} and size ratio q, and reads

$$\alpha_{\mathrm{FVT}} = (1 - \eta_{\mathrm{c}}) \exp\left[-A\gamma - B\gamma^2 - C\gamma^3\right], \tag{4}$$

with $\gamma = \eta_{\mathrm{c}}/(1 - \eta_{\mathrm{c}})$, $A = 3q + 3q^2 + q^3$, $B = 9q^2/2 + 3q^3$ and $C = 3q^3$. Here we select a value of $\eta_{\mathrm{p}}^{\mathrm{r}} = 0.5$.

We start by computing the effective two-body potential of mean force (PMF) $\Phi^{(2)}$ in the fine-grained (FG) PAO model by performing canonical constraint MD (CMD-NVT) simulations as described in the main text. In particular, we sample the mean forces acting on the colloids at 219 separation distances in the range $0.98 \leq R_{ij}/\sigma_{\mathrm{c}} \leq 2.50$. Each MD simulation, consists of 5×10^8 steps, where we collect the instantaneous forces on the colloids every 1000 steps. In all the MD simulations, we set the mass m of the colloid and polymers

to unity and use a timestep of $\delta t = 0.001\tau$, with $\tau = \sqrt{\epsilon/(m\sigma^2)}$. Furthermore, we set the temperature at $T = 1.5\epsilon/k_B$. The two-body potential of mean force $\Phi^{(2)}$ as obtained by numerical integration of the mean forces (Eq. 10 of the main text) is reported in Fig. S1(a), where, for comparison purposes, we also include the exact AO depletion pair potential for two colloids immersed in a suspension of non-adsorbing polymers (depletants) with $q = 1.0$ and at a reservoir polymer packing fraction $\eta_{\mathrm{p}}^{\mathrm{r}} = 0.5$. The AO depletion pair potential reads

$$\beta\phi_{\mathrm{AO}}(R_{ij}) = \begin{cases} -\eta_{\mathrm{p}}^{\mathrm{r}} \frac{(1+q)^3}{q^3} \left[1 - \frac{3R_{ij}}{2(1+q)\sigma_{\mathrm{c}}} + \frac{R_{ij}^3}{2(1+q)^3\sigma_{\mathrm{c}}^3}\right] & \text{for } \sigma_{\mathrm{c}} < R_{ij} < 2\sigma_{\mathrm{cp}} \\ 0 & \text{for } R_{ij} > 2\sigma_{\mathrm{cp}}, \end{cases} \tag{5}$$

with $\beta = (k_{\mathrm{B}}T)^{-1}$, the inverse temperature.

We observe an excellent correspondence between the true AO pair potential and the PMF in the PAO model over the whole range of separation distances. This shows that the pseudo hard-sphere potential accurately mimics the hard-core repulsion of the true hard-sphere model, and therefore exhibits the well-known depletion interaction of attractive nature between the colloids. However, we note that for the parameters considered, the minimum in the original AO pair potential occurs at contact ($R_{ij} = \sigma_{\mathrm{c}}$) with a strength of $-1.25k_{\mathrm{B}}T$, while in the PAO model, the minimum of $\sim -1.23k_{\mathrm{B}}T$ occurs at $R_{ij} = 1.01\sigma_{\mathrm{c}}$.

As in the case of the ligand-stabilized nanoparticles discussed in the main text, we proceed to fit directly the vectorial components of the mean forces acting on the center-of-masses of the two individual colloid cores i and j for each separation distance (a total of 657 force components per particle). The fitting is performed using only gradients of radial symmetry functions (SFs).The parameters in the initial pool are: $\mu/\sigma_{\mathrm{c}}^{-2} = \{0.001, 1.715214286, 3.429428571, 5.143642857, 6.857857143, 8.572071429, 10.28628571, 12.0005, 15.42892857\}$ and $R_s/\sigma_{\mathrm{c}} = \{0.0, 0.1, 0.2, 0.3, 0.4, 0.5, 0.6, 0.7, 0.8, 0.9\}$. We use a single cut-off value of $R_c = 2.0\sigma_{\mathrm{cp}}$. The ML potential, which we will refer to as ML2 potential, is constructed with solely $N_{SF} = 18$ gradients of radial SFs and presents a correlation coefficient $R^2 \approx 0.998$ and Root Mean Square Error (RMSE) of $0.0304k_{\mathrm{B}}T/\sigma_c$. A parity plot comparing the ML predicted and reference

FG mean forces is shown in Fig. S1(b). To test the model, we evaluate the effective potential between a pair of colloids at varying separation distances using the model constructed by directly fitting the two-body mean forces ($\Phi^{(2)}_{\mathrm{ML2}}(R_{ij})$) and plot the results in Fig. S1(a). We observe a very good quantitative agreement between the reference PAO and ML2 models.

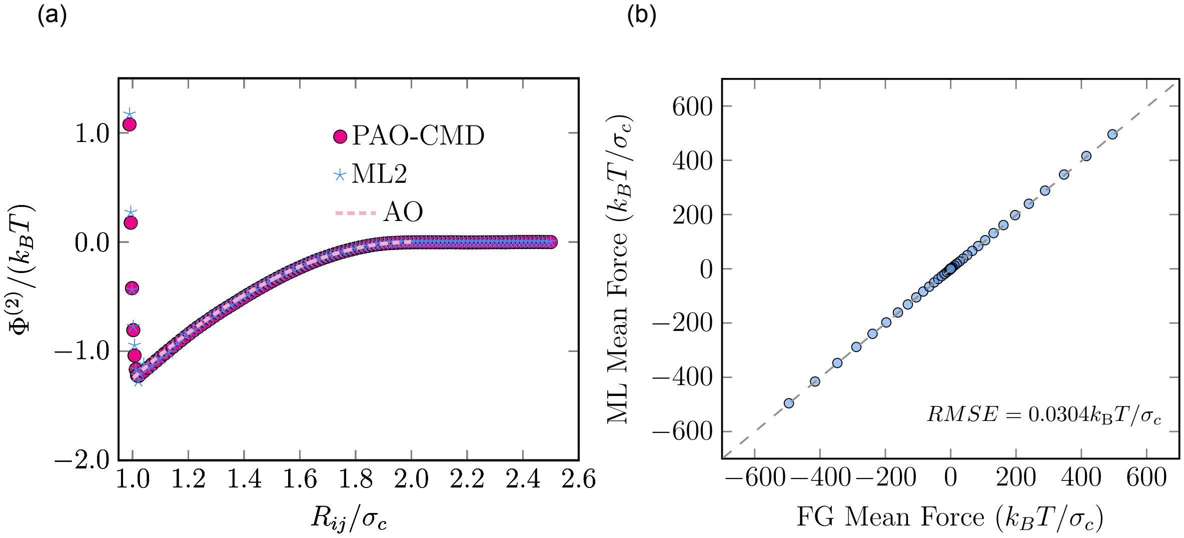

Fig. S1: (a) Effective two-body interaction potential between colloids of diameter σ_{c} as a function of their separation distance R_{ij} in a suspension containing non-adsorbing polymers of diameter $\sigma_{\mathrm{p}} = \sigma_{\mathrm{c}}$ at a reservoir polymer packing fraction $\eta^{\mathrm{r}}_{\mathrm{p}} = 0.5$. Solid circles correspond to the values obtained by numerical integration of the mean forces in the PAO model, while the stars represent the values predicted by the ML2 model trained with the mean forces. The exact depletion AO pair potential (Eq. 5) for the same parameters is indicated with the dashed lines. (b) Parity plot showing the vectorial components of the mean forces measured in the FG model and those predicted by the ML2 model.

As discussed above, for the model at hand, one can expect many-body effects to become largely pronounced, especially at relatively large colloid packing fractions and high polymer concentrations.[3,12] Because of geometrical arguments, in the original AO model, a formal decomposition of the total effective interactions in the one-component model into zero-, one-, ..., $k-$body contributions can be achieved, which then allows one to evaluate the individual terms by numerical methods.[3,12] We can also construct an effective colloids-only potential for the PAO model incorporating many-body effects using our multiscale ML method. To

this end, we first perform a number of reference NVT-MD simulations of systems containing $N_{\mathrm{c}} = 108$ colloids in the packing fraction range $0.0157 \leq \eta_{\mathrm{c}} \leq 0.1012$. In order to generate a large number of diverse local configurations for the colloids, we perform equlibrium MD simulations at four different packing fractions, namely $\eta_{\mathrm{p}}^{\mathrm{r}} = 0.5$, 5, 7 and 10. As above, we determine the number of polymer coils in the NVT simulations using Eq. 3. Depending on the value of $\eta_{\mathrm{p}}^{\mathrm{r}}$, which determines the strength of the interactions, as well as on η_{c}, colloidal particles can be found isolated (far from first neighbors), forming small clusters of varying size or even assembled into finite size crystallites. From all the generated MD trajectories, we then select a number of colloid configurations that we subsequently use as the initial state for CMD simulations where we sample the mean forces on the colloids at $\eta_{\mathrm{p}}^{\mathrm{r}} = 0.5$. Overall, we consider a dataset containing a total of 28,200 vectorial forces and perform a linear regression using both gradients of radial and angular SFs. In the candidate pool of gradients of SFs, the radial functions are generated using the same parameters used when constructing the ML2 model and we consider $\mu_a/\sigma_{\mathrm{c}}^{-2} = \{0.001, 0.01, 0.1, 1, 2, 4, 8\}$, $\xi = \{1.0, 2.0, 4.0, 8.0\}$ and $\lambda = \{-1, 1\}$ for the angular functions. A closed solution with $N_{SF} = 135$ yields an optimal model for the many-body mean forces with a correlation coefficient of $R^2 \approx 0.998$ and a $RMSE \approx 5.602 k_B T/\sigma_{\mathrm{c}}$. We will refer to the so-obtained model as the many-body potential ML108 ($\Phi_{\mathrm{ML108}}(\boldsymbol{R}^{108})$).

As described above, in the AO model, the strength of the depletant-mediated total effective interactions between the colloids scales linearly with $\eta_{\mathrm{p}}^{\mathrm{r}}$. Thus, in the limit of $\eta_{\mathrm{p}}^{\mathrm{r}} \to 0$ one naturally recovers the hard-sphere interactions between colloids, whereas at high $\eta_{\mathrm{p}}^{\mathrm{r}}$, the balance of two- and many-body contributions determine the resulting interactions. While in the one-component AO model the two-body polymer-induced interaction is attractive, the many-body interactions lead to a purely repulsive contribution to the total potential.[3] Indeed, one of the main effects of many-body contributions in the colloid-polymer mixture of size ratio $q = 1$ is to shift the critical point of the gas-liquid coexistence.[3,12] In order to compare the two-body potential Φ_{ML2} and many-body potential Φ_{ML108}, and to asses the

effect of many-body interactions, we perform Monte Carlo (MC) simulations of bulk systems of $N = 256$ colloids at a high packing fraction where many-body contributions arise, namely $\eta_c = 0.45$.[3,12] We then fix $\eta_p^r = 0.5$ and $\eta_p^r = 2.0$ and sample the pair-correlation functions of the colloidal particles, $g_{cc}(R_{ij})$. We note that since we have constructed CG colloids-only models for the binary PAO system at $\eta_p^r = 0.5$, we can directly transfer them to other state points by scaling with a factor $\zeta \equiv \eta_p^r/0.5$. In Fig. 8(a) and 8(b) in the main text, we report the colloid-colloid pair correlation functions measured in the PAO FG and CG ML2 and ML108 models at the two different states. At $\eta_p^r = 0.5$, both the ML2 and ML108 closely match the fluid-like structure of the FG model. At $\eta_p^r = 2.0$, the FG model still exhibits a fluid-like structure, which is well reproduced by the CG ML108 potential. Conversely, the CG ML2 potential leads to an equilibrium state exhibiting an ordered face-centered-cubic (FCC) structure. Such a big difference is the result of an overestimation of the cohesive (attractive) depletion interaction in the ML2 potential. The clear mismatch in the structure of the systems described by the CG ML2 and ML108 potentials (see also Fig. 8(c) in the main text) evidences the importance of the many-body interactions.

References

(1) Dijkstra, M.; Brader, J. M.; Evans, R. Phase behaviour and structure of model colloid-polymer mixtures. *Journal of Physics: Condensed Matter* **1999**, *11*, 10079.

(2) Vink, R.; Horbach, J. Grand canonical Monte Carlo simulation of a model colloid–polymer mixture: Coexistence line, critical behavior, and interfacial tension. *The Journal of chemical physics* **2004**, *121*, 3253–3258.

(3) Dijkstra, M.; van Roij, R.; Roth, R.; Fortini, A. Effect of many-body interactions on the bulk and interfacial phase behavior of a model colloid-polymer mixture. *Physical Review E* **2006**, *73*, 041404.

(4) Verso, F. L.; Vink, R.; Pini, D.; Reatto, L. Critical behavior in colloid-polymer mixtures: Theory and simulation. *Physical Review E* **2006**, *73*, 061407.

(5) Asakura, S.; Oosawa, F. On interaction between two bodies immersed in a solution of macromolecules. *The Journal of chemical physics* **1954**, *22*, 1255–1256.

(6) Asakura, S.; Oosawa, F. Interaction between particles suspended in solutions of macromolecules. *Journal of polymer science* **1958**, *33*, 183–192.

(7) Vrij, A. Polymers at interfaces and the interactions in colloidal dispersions. *Pure and Applied Chemistry* **1976**, *48*, 471–483.

(8) Jover, J.; Haslam, A.; Galindo, A.; Jackson, G.; Müller, E. Pseudo hard-sphere potential for use in continuous molecular-dynamics simulation of spherical and chain molecules. *The Journal of chemical physics* **2012**, *137*, 144505.

(9) Thompson, A. P.; Aktulga, H. M.; Berger, R.; Bolintineanu, D. S.; Brown, W. M.; Crozier, P. S.; in't Veld, P. J.; Kohlmeyer, A.; Moore, S. G.; Nguyen, T. D., et al. LAMMPS-a flexible simulation tool for particle-based materials modeling at the atomic, meso, and continuum scales. *Computer Physics Communications* **2022**, *271*, 108171.

(10) Gast, A.; Hall, C.; Russel, W. Polymer-induced phase separations in nonaqueous colloidal suspensions. *Journal of Colloid and Interface Science* **1983**, *96*, 251–267.

(11) Lekkerkerker, H. N.; Poon, W.-K.; Pusey, P. N.; Stroobants, A.; Warren, P. . Phase behaviour of colloid+ polymer mixtures. *Europhysics Letters* **1992**, *20*, 559.

(12) Campos-Villalobos, G.; Boattini, E.; Filion, L.; Dijkstra, M. Machine learning many-body potentials for colloidal systems. *The Journal of Chemical Physics* **2021**, *155*, 174902.
